# (2*S*)-3-(1*H*-Indol-3-yl)-2-(4-methyl­benzene­sulfonamido)­propionic acid monohydrate

**DOI:** 10.1107/S1600536811032089

**Published:** 2011-08-11

**Authors:** Islam Ullah Khan, Muhammad Nadeem Arshad, Hafiz Mubashar-ur-Rehman, William T. A. Harrison, Muhammad Baqir Ali

**Affiliations:** a Materials Chemistry Laboratory, Department of Chemistry, GC University, Lahore 54000, Pakistan; b Department of Chemistry, University of Aberdeen, Aberdeen, AB24 3UE, Scotland

## Abstract

In the title compound, C_18_H_18_N_2_O_4_S·H_2_O, the indole and toluene ring systems are oriented at a dihedral angle of 84.51 (9)°. In the crystal, the components are linked by N—H⋯O, O—H⋯O, C—H⋯O and N—H⋯π inter­actions. These include a short link from the α-C atom of the amino acid fragment.

## Related literature

For details of the synthesis, see: Deng & Mani (2006 ▶). For background to sulfonamides in biology, see: Parka *et al.* (2009 ▶); Wang *et al.* (2007 ▶). For related structures, see: Li *et al.* (2008 ▶); Khan *et al.* (2011 ▶).
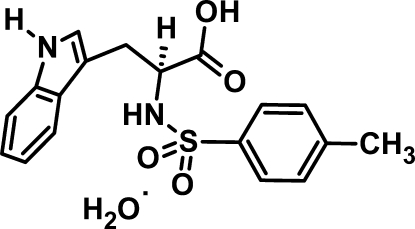


## Experimental

### 

#### Crystal data

C_18_H_18_N_2_O_4_S·H_2_O
*M* *_r_* = 376.42
Monoclinic, 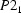

*a* = 8.4531 (10) Å
*b* = 5.2521 (5) Å
*c* = 20.867 (2) Å
β = 98.056 (4)°
*V* = 917.30 (17) Å^3^
*Z* = 2
Mo *K*α radiation
μ = 0.21 mm^−1^
*T* = 296 K
0.28 × 0.11 × 0.09 mm

#### Data collection

Bruker Kappa APEXII CCD diffractometer
Absorption correction: multi-scan (*SADABS*; Bruker, 2007 ▶) *T*_min_ = 0.944, *T*_max_ = 0.982
10939 measured reflections
4475 independent reflections
2135 reflections with *I* > 2σ(*I*)
*R*_int_ = 0.064

#### Refinement

*R*[*F* ^2^ > 2σ(*F* ^2^)] = 0.055
*wR*(*F* ^2^) = 0.107
*S* = 0.94
4475 reflections
243 parameters
1 restraint
H atoms treated by a mixture of independent and constrained refinement
Δρ_max_ = 0.20 e Å^−3^
Δρ_min_ = −0.25 e Å^−3^
Absolute structure: Flack (1983 ▶), 1951 Friedel pairs
Flack parameter: −0.05 (10)

### 

Data collection: *APEX2* (Bruker, 2007 ▶); cell refinement: *SAINT* (Bruker, 2007 ▶); data reduction: *SAINT*; program(s) used to solve structure: *SHELXS97* (Sheldrick, 2008 ▶); program(s) used to refine structure: *SHELXL97* (Sheldrick, 2008 ▶); molecular graphics: *ORTEP-3* (Farrugia, 1997) ▶ and *PLATON* (Spek, 2009 ▶); software used to prepare material for publication: *WinGX* (Farrugia, 1999 ▶) and *PLATON*.

## Supplementary Material

Crystal structure: contains datablock(s) I, global. DOI: 10.1107/S1600536811032089/wn2443sup1.cif

Structure factors: contains datablock(s) I. DOI: 10.1107/S1600536811032089/wn2443Isup2.hkl

Supplementary material file. DOI: 10.1107/S1600536811032089/wn2443Isup3.cml

Additional supplementary materials:  crystallographic information; 3D view; checkCIF report

## Figures and Tables

**Table 1:** Hydrogen-bond geometry (Å, °) *Cg*3 is the centroid of the C12–C17 ring.

*D*—H⋯*A*	*D*—H	H⋯*A*	*D*⋯*A*	*D*—H⋯*A*
N1—H1*N*⋯O2^i^	0.88 (4)	2.37 (4)	3.208 (4)	160 (3)
N2—H2*N*⋯*Cg*3^ii^	0.79 (4)	2.85 (4)	3.480 (4)	139 (4)
O3—H3*O*⋯O5^iii^	0.82	1.81	2.629 (4)	177
C7—H7⋯O4^iv^	0.98	2.37	3.205 (4)	143
C18—H18*C*⋯O1^v^	0.96	2.59	3.456 (5)	151
O5—H1*W*⋯O2^i^	0.89	2.05	2.935 (4)	174

Symmetry codes: (i) 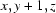; (ii) 
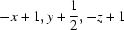
; (iii) 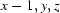; (iv) 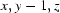; (v) 
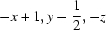
.

**Fig. 1.**
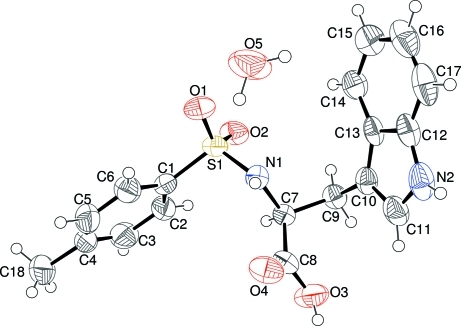
The molecular structure of the title compound, with displacement ellipsoids drawn at the 50% probability level. Hydrogen atoms are shown as spheres of arbitrary radius.

**Fig. 2.**
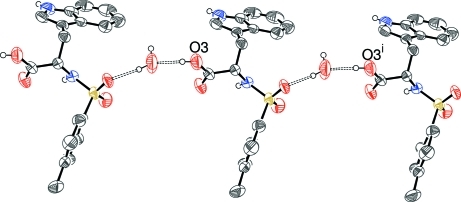
Partial packing diagram, showing [110] chains of alternating organic and water molecules linked by O—H···O hydrogen bonds shown as dashed lines. Symmetry code: (i) x+1, y–1, z.

## supplementary crystallographic information

### Comment

Tryptophan-based sulfonamides have been reported as non-hydroxamate TNF-α converting enzyme (TACE) inhibitors (Parka *et al.*, 2009), and in another study (Wang *et al.*, 2007) some are reported as acetohydroxy acid synthase (AHAS) inhibitors. The previously reported crystal structures of two active compounds, namely (*S*)-methyl 2-(4-R-phenylsulfonamido)-3- (1*H*-indol-3-yl)propanoate [R = H  and Cl] (Li *et al.*, 2008) are closely related to the title compound.

In the crystal structure, a water molecule crystallized as a solvent of crystallization along with the sulfonamide (Fig. 1). The indole system (C10—C17/N2) and aromatic ring (C1—C6) are inclined to each other at a dihedral angle of 84.51 (9)°. The plane of the carboxylic acid group is twisted at dihedral angles of  71.46 (13)° and 63.62 (9)° with respect to the aromatic ring and indole unit, respectively.

The configuration of the stereogenic carbon atom, C7, is S, which is consistent with that of the equivalent atom in the starting material.

In the crystal structure, the components are linked by a variety of interactions (Table 1).  The carboxylic acid makes an O—H···O hydrogen bond to the water molecule, and the water molecule is involved in the same type of hydrogen bond to the sulfonyl group, to generate alternating [110] chains of the two species. The amino-acid N—H group forms an intermolecular link to the sulfonyl group. The indole N—H group forms an N—H···π bond to the six-membered ring of the indole system of an adjacent molecule. Two C—H···O interactions are also present; a strong link from the α-carbon atom, C7, as also seen in related structures (Khan *et al.*, 2011) and a weaker link from the methyl group.

### Experimental

The title compound was prepared following the literature method (Deng & Mani, 2006) and recrystalized from methanol by slow evaporation to yield colourless needles.

### Refinement

The C-bound H-atoms were positioned with idealized geometry with C—H = 0.93 Å for aromatic,  C—H = 0.96 Å for methyl, C—H = 0.97 Å for methylene, C—H = 0.98 Å for methine, and were refined using a riding model with *U*_iso_(H) = 1.2 *U*_eq_(C) but *U*_iso_(H) = 1.5 *U*_eq_(C) for methyl.

The hydroxyl H-atom of the carboxylic acid group was also positioned with idealized geometry, O—H = 0.82 Å, and refined as riding with *U*_iso_(H) = 1.5 *U*_eq_(O).

The H atoms of the  water molecule were located in a difference map with O—H = 0.893–0.900Å, and refined as riding with *U*_iso_(H) = *U*_eq_(O).

The H atoms attached to N were located in a difference map and refined freeely.

### Crystal data

**Table:**

C_18_H_18_N_2_O_4_S·H_2_O	*F*(000) = 396
*M**_r_* = 376.42	*D*_x_ = 1.363 Mg m^−^^3^
Monoclinic, *P*2_1_	Mo *K*α radiation, λ = 0.71073 Å
Hall symbol: P 2yb	Cell parameters from 1381 reflections
*a* = 8.4531 (10) Å	θ = 2.4–19.2°
*b* = 5.2521 (5) Å	µ = 0.21 mm^−^^1^
*c* = 20.867 (2) Å	*T* = 296 K
β = 98.056 (4)°	Needle, colorless
*V* = 917.30 (17) Å^3^	0.28 × 0.11 × 0.09 mm
*Z* = 2	

### Data collection

**Table:**

Bruker Kappa APEXII CCD diffractometer	4475 independent reflections
Radiation source: fine-focus sealed tube	2135 reflections with *I* > 2σ(*I*)
graphite	*R*_int_ = 0.064
φ and ω scans	θ_max_ = 28.3°, θ_min_ = 2.4°
Absorption correction: multi-scan (*SADABS*; Bruker, 2007)	*h* = −11→10
*T*_min_ = 0.944, *T*_max_ = 0.982	*k* = −6→7
10939 measured reflections	*l* = −27→27

### Refinement

**Table:**

Refinement on *F*^2^	Secondary atom site location: difference Fourier map
Least-squares matrix: full	Hydrogen site location: inferred from neighbouring sites
*R*[*F*^2^ > 2σ(*F*^2^)] = 0.055	H atoms treated by a mixture of independent and constrained refinement
*wR*(*F*^2^) = 0.107	*w* = 1/[σ^2^(*F*_o_^2^) + (0.034*P*)^2^] where *P* = (*F*_o_^2^ + 2*F*_c_^2^)/3
*S* = 0.94	(Δ/σ)_max_ < 0.001
4475 reflections	Δρ_max_ = 0.20 e Å^−^^3^
243 parameters	Δρ_min_ = −0.25 e Å^−^^3^
1 restraint	Absolute structure: Flack (1983), 1951 Friedel pairs
Primary atom site location: structure-invariant direct methods	Flack parameter: −0.05 (10)

### Special details

**Table:**

Geometry. All s.u.'s (except the s.u. in the dihedral angle between two l.s. planes) are estimated using the full covariance matrix. The cell s.u.'s are taken into account individually in the estimation of s.u.'s in distances, angles and torsion angles; correlations between s.u.'s in cell parameters are only used when they are defined by crystal symmetry. An approximate (isotropic) treatment of cell s.u.'s is used for estimating s.u.'s involving l.s. planes.
Refinement. Refinement of F^2^ against ALL reflections. The weighted R-factor wR and goodness of fit S are based on F^2^, conventional R-factors R are based on F, with F set to zero for negative F^2^. The threshold expression of F^2^ > 2σ(F^2^) is used only for calculating R-factors(gt) etc. and is not relevant to the choice of reflections for refinement. R-factors based on F^2^ are statistically about twice as large as those based on F, and R- factors based on ALL data will be even larger.

### Fractional atomic coordinates and isotropic or equivalent isotropic displacement parameters (Å^2^)

**Table:**

	*x*	*y*	*z*	*U*_iso_*/*U*_eq_	
C1	0.3845 (4)	0.3366 (8)	0.10176 (15)	0.0401 (8)	
C2	0.2730 (4)	0.1527 (8)	0.08284 (18)	0.0527 (10)	
H2	0.2528	0.0243	0.1113	0.063*	
C3	0.1902 (5)	0.1612 (9)	0.0202 (2)	0.0679 (12)	
H3	0.1150	0.0358	0.0069	0.081*	
C4	0.2171 (5)	0.3511 (11)	−0.02263 (17)	0.0619 (11)	
C5	0.3258 (5)	0.5359 (10)	−0.0017 (2)	0.0675 (12)	
H5	0.3435	0.6676	−0.0296	0.081*	
C6	0.4098 (4)	0.5318 (8)	0.05980 (19)	0.0561 (10)	
H6	0.4832	0.6597	0.0731	0.067*	
C7	0.2547 (4)	0.3994 (6)	0.24734 (14)	0.0352 (8)	
H7	0.2140	0.2592	0.2187	0.042*	
C8	0.1348 (5)	0.6132 (8)	0.23914 (16)	0.0452 (10)	
C9	0.2837 (4)	0.2991 (7)	0.31717 (14)	0.0450 (9)	
H9A	0.1823	0.2471	0.3298	0.054*	
H9B	0.3516	0.1498	0.3187	0.054*	
C10	0.3599 (5)	0.4909 (7)	0.36487 (16)	0.0454 (9)	
C11	0.2860 (5)	0.6835 (8)	0.39356 (18)	0.0585 (11)	
H11	0.1769	0.7169	0.3868	0.070*	
C12	0.5453 (6)	0.7203 (8)	0.43133 (18)	0.0552 (11)	
C13	0.5242 (5)	0.5127 (7)	0.38869 (15)	0.0451 (9)	
C14	0.6611 (5)	0.3742 (8)	0.37809 (17)	0.0539 (10)	
H14	0.6522	0.2370	0.3497	0.065*	
C15	0.8074 (6)	0.4441 (8)	0.4102 (2)	0.0704 (13)	
H15	0.8977	0.3517	0.4037	0.084*	
C16	0.8233 (6)	0.6528 (10)	0.4526 (2)	0.0782 (14)	
H16	0.9239	0.6974	0.4736	0.094*	
C17	0.6930 (6)	0.7908 (9)	0.46353 (18)	0.0725 (14)	
H17	0.7031	0.9285	0.4918	0.087*	
C18	0.1307 (5)	0.3506 (12)	−0.09171 (16)	0.0977 (16)	
H18A	0.0896	0.5178	−0.1027	0.147*	
H18B	0.0439	0.2311	−0.0952	0.147*	
H18C	0.2039	0.3026	−0.1208	0.147*	
S1	0.49606 (10)	0.32979 (18)	0.17916 (4)	0.0446 (2)	
N1	0.4035 (3)	0.4923 (6)	0.22720 (13)	0.0402 (7)	
H1N	0.413 (4)	0.659 (7)	0.2271 (15)	0.048*	
N2	0.3962 (5)	0.8178 (7)	0.43322 (15)	0.0681 (11)	
H2N	0.375 (5)	0.941 (8)	0.452 (2)	0.082*	
O1	0.6432 (3)	0.4579 (5)	0.17601 (12)	0.0647 (8)	
O2	0.4980 (3)	0.0709 (4)	0.20122 (12)	0.0553 (7)	
O3	−0.0007 (3)	0.5429 (6)	0.25925 (15)	0.0744 (8)	
H3O	−0.0566	0.6686	0.2623	0.112*	
O4	0.1563 (3)	0.8175 (5)	0.21751 (12)	0.0623 (7)	
O5	0.8218 (3)	0.9515 (6)	0.26435 (15)	0.1120 (13)	
H1W	0.7260	0.9876	0.2425	0.134*	
H2W	0.8393	0.9747	0.3075	0.134*	

### Atomic displacement parameters (Å^2^)

**Table:**

	*U*^11^	*U*^22^	*U*^33^	*U*^12^	*U*^13^	*U*^23^
C1	0.0351 (19)	0.042 (2)	0.0446 (19)	0.002 (2)	0.0098 (15)	0.000 (2)
C2	0.048 (3)	0.061 (3)	0.048 (2)	−0.004 (2)	0.004 (2)	0.000 (2)
C3	0.051 (3)	0.087 (4)	0.063 (3)	−0.008 (3)	0.000 (2)	−0.018 (3)
C4	0.058 (3)	0.090 (3)	0.039 (2)	0.032 (3)	0.009 (2)	−0.003 (3)
C5	0.075 (3)	0.075 (3)	0.054 (3)	0.010 (3)	0.018 (3)	0.015 (3)
C6	0.054 (3)	0.057 (3)	0.057 (3)	−0.004 (2)	0.006 (2)	0.007 (2)
C7	0.036 (2)	0.030 (2)	0.0386 (19)	−0.0023 (15)	0.0010 (15)	−0.0027 (14)
C8	0.044 (3)	0.043 (2)	0.045 (2)	0.0024 (19)	−0.0057 (19)	0.005 (2)
C9	0.052 (2)	0.036 (2)	0.047 (2)	0.0024 (19)	0.0053 (17)	0.0048 (19)
C10	0.063 (3)	0.036 (2)	0.037 (2)	0.0040 (19)	0.009 (2)	0.0035 (18)
C11	0.075 (3)	0.054 (3)	0.044 (2)	0.011 (2)	0.000 (2)	0.010 (2)
C12	0.083 (3)	0.045 (2)	0.036 (2)	−0.002 (2)	0.001 (2)	0.0037 (18)
C13	0.071 (3)	0.035 (2)	0.0279 (19)	0.006 (2)	0.0029 (19)	0.0029 (17)
C14	0.064 (3)	0.048 (3)	0.048 (2)	−0.004 (2)	0.003 (2)	−0.005 (2)
C15	0.070 (3)	0.077 (3)	0.064 (3)	−0.001 (2)	0.007 (2)	−0.001 (2)
C16	0.095 (4)	0.081 (4)	0.053 (3)	−0.030 (3)	−0.008 (3)	0.002 (3)
C17	0.121 (4)	0.053 (3)	0.041 (2)	−0.021 (3)	0.003 (3)	−0.004 (2)
C18	0.091 (3)	0.161 (5)	0.040 (2)	0.040 (4)	0.006 (2)	−0.013 (3)
S1	0.0341 (5)	0.0464 (6)	0.0523 (5)	0.0018 (5)	0.0027 (4)	0.0029 (6)
N1	0.0448 (19)	0.0351 (17)	0.0408 (17)	−0.0059 (14)	0.0063 (14)	−0.0042 (15)
N2	0.115 (3)	0.045 (2)	0.044 (2)	0.018 (3)	0.008 (2)	−0.006 (2)
O1	0.0332 (16)	0.084 (2)	0.0765 (19)	−0.0112 (14)	0.0066 (14)	0.0007 (16)
O2	0.0540 (18)	0.0403 (16)	0.0704 (17)	0.0153 (13)	0.0050 (14)	0.0139 (14)
O3	0.0496 (19)	0.077 (2)	0.100 (2)	0.0180 (15)	0.0216 (17)	0.0269 (19)
O4	0.0631 (17)	0.0382 (15)	0.0824 (17)	0.0088 (18)	−0.0015 (13)	0.0116 (18)
O5	0.081 (2)	0.143 (3)	0.104 (2)	0.065 (2)	−0.0165 (19)	−0.035 (2)

### Geometric parameters (Å, °)

**Table:**

C1—C2	1.369 (5)	C11—H11	0.9300
C1—C6	1.384 (5)	C12—N2	1.366 (5)
C1—S1	1.753 (3)	C12—C17	1.383 (6)
C2—C3	1.395 (5)	C12—C13	1.403 (5)
C2—H2	0.9300	C13—C14	1.411 (5)
C3—C4	1.379 (6)	C14—C15	1.372 (5)
C3—H3	0.9300	C14—H14	0.9300
C4—C5	1.366 (6)	C15—C16	1.404 (6)
C4—C18	1.522 (5)	C15—H15	0.9300
C5—C6	1.377 (5)	C16—C17	1.365 (6)
C5—H5	0.9300	C16—H16	0.9300
C6—H6	0.9300	C17—H17	0.9300
C7—N1	1.465 (4)	C18—H18A	0.9600
C7—C8	1.506 (5)	C18—H18B	0.9600
C7—C9	1.536 (4)	C18—H18C	0.9600
C7—H7	0.9800	S1—O1	1.423 (2)
C8—O4	1.188 (4)	S1—O2	1.435 (2)
C8—O3	1.327 (4)	S1—N1	1.601 (3)
C9—C10	1.497 (5)	N1—H1N	0.88 (4)
C9—H9A	0.9700	N2—H2N	0.79 (4)
C9—H9B	0.9700	O3—H3O	0.8200
C10—C11	1.370 (5)	O5—H1W	0.8926
C10—C13	1.412 (5)	O5—H2W	0.9002
C11—N2	1.354 (5)		
			
C2—C1—C6	120.1 (3)	N2—C12—C17	131.2 (4)
C2—C1—S1	120.7 (3)	N2—C12—C13	105.9 (4)
C6—C1—S1	119.2 (3)	C17—C12—C13	122.8 (4)
C1—C2—C3	118.9 (4)	C12—C13—C14	117.8 (4)
C1—C2—H2	120.6	C12—C13—C10	108.5 (4)
C3—C2—H2	120.6	C14—C13—C10	133.7 (3)
C4—C3—C2	121.6 (4)	C15—C14—C13	119.3 (4)
C4—C3—H3	119.2	C15—C14—H14	120.3
C2—C3—H3	119.2	C13—C14—H14	120.3
C5—C4—C3	118.1 (4)	C14—C15—C16	121.1 (4)
C5—C4—C18	121.1 (5)	C14—C15—H15	119.4
C3—C4—C18	120.8 (5)	C16—C15—H15	119.4
C4—C5—C6	121.5 (4)	C17—C16—C15	120.8 (5)
C4—C5—H5	119.2	C17—C16—H16	119.6
C6—C5—H5	119.2	C15—C16—H16	119.6
C5—C6—C1	119.7 (4)	C16—C17—C12	118.2 (5)
C5—C6—H6	120.1	C16—C17—H17	120.9
C1—C6—H6	120.1	C12—C17—H17	120.9
N1—C7—C8	108.1 (3)	C4—C18—H18A	109.5
N1—C7—C9	110.9 (3)	C4—C18—H18B	109.5
C8—C7—C9	112.2 (3)	H18A—C18—H18B	109.5
N1—C7—H7	108.5	C4—C18—H18C	109.5
C8—C7—H7	108.5	H18A—C18—H18C	109.5
C9—C7—H7	108.5	H18B—C18—H18C	109.5
O4—C8—O3	123.8 (4)	O1—S1—O2	119.49 (16)
O4—C8—C7	125.5 (4)	O1—S1—N1	106.46 (16)
O3—C8—C7	110.7 (3)	O2—S1—N1	106.77 (15)
C10—C9—C7	113.4 (3)	O1—S1—C1	107.97 (16)
C10—C9—H9A	108.9	O2—S1—C1	107.19 (18)
C7—C9—H9A	108.9	N1—S1—C1	108.60 (15)
C10—C9—H9B	108.9	C7—N1—S1	121.1 (2)
C7—C9—H9B	108.9	C7—N1—H1N	114 (2)
H9A—C9—H9B	107.7	S1—N1—H1N	119 (2)
C11—C10—C13	105.8 (3)	C11—N2—C12	110.2 (4)
C11—C10—C9	127.4 (4)	C11—N2—H2N	123 (3)
C13—C10—C9	126.8 (3)	C12—N2—H2N	126 (3)
N2—C11—C10	109.6 (4)	C8—O3—H3O	109.5
N2—C11—H11	125.2	H1W—O5—H2W	119.5
C10—C11—H11	125.2		
			
C6—C1—C2—C3	2.2 (5)	C9—C10—C13—C12	178.8 (3)
S1—C1—C2—C3	−178.1 (3)	C11—C10—C13—C14	180.0 (4)
C1—C2—C3—C4	−0.6 (6)	C9—C10—C13—C14	−1.0 (6)
C2—C3—C4—C5	−1.2 (6)	C12—C13—C14—C15	1.0 (5)
C2—C3—C4—C18	177.4 (4)	C10—C13—C14—C15	−179.2 (4)
C3—C4—C5—C6	1.5 (6)	C13—C14—C15—C16	−0.8 (6)
C18—C4—C5—C6	−177.1 (4)	C14—C15—C16—C17	0.5 (6)
C4—C5—C6—C1	0.0 (6)	C15—C16—C17—C12	−0.4 (6)
C2—C1—C6—C5	−2.0 (5)	N2—C12—C17—C16	178.8 (4)
S1—C1—C6—C5	178.4 (3)	C13—C12—C17—C16	0.6 (6)
N1—C7—C8—O4	2.9 (5)	C2—C1—S1—O1	153.8 (3)
C9—C7—C8—O4	125.5 (4)	C6—C1—S1—O1	−26.6 (3)
N1—C7—C8—O3	−177.4 (3)	C2—C1—S1—O2	23.8 (3)
C9—C7—C8—O3	−54.8 (4)	C6—C1—S1—O2	−156.5 (3)
N1—C7—C9—C10	55.8 (4)	C2—C1—S1—N1	−91.2 (3)
C8—C7—C9—C10	−65.2 (4)	C6—C1—S1—N1	88.5 (3)
C7—C9—C10—C11	82.0 (4)	C8—C7—N1—S1	−132.1 (3)
C7—C9—C10—C13	−96.8 (4)	C9—C7—N1—S1	104.5 (3)
C13—C10—C11—N2	−0.3 (4)	O1—S1—N1—C7	−173.1 (3)
C9—C10—C11—N2	−179.3 (3)	O2—S1—N1—C7	−44.4 (3)
N2—C12—C13—C14	−179.5 (3)	C1—S1—N1—C7	70.8 (3)
C17—C12—C13—C14	−0.9 (5)	C10—C11—N2—C12	0.7 (4)
N2—C12—C13—C10	0.6 (4)	C17—C12—N2—C11	−179.3 (4)
C17—C12—C13—C10	179.2 (4)	C13—C12—N2—C11	−0.8 (4)
C11—C10—C13—C12	−0.2 (4)		

### Hydrogen-bond geometry (Å, °)

**Table:**

Cg3 is the centroid of the C12–C17 ring.

**Table:**

*D*—H···*A*	*D*—H	H···*A*	*D*···*A*	*D*—H···*A*
N1—H1N···O2^i^	0.88 (4)	2.37 (4)	3.208 (4)	160 (3)
N2—H2N···Cg3^ii^	0.79 (4)	2.85 (4)	3.480 (4)	139 (4)
O3—H3O···O5^iii^	0.82	1.81	2.629 (4)	177
C7—H7···O4^iv^	0.98	2.37	3.205 (4)	143
C18—H18C···O1^v^	0.96	2.59	3.456 (5)	151
O5—H1W···O2^i^	0.89	2.05	2.935 (4)	174

Symmetry codes: (i) *x*, *y*+1, *z*; (ii) −*x*+1, *y*+1/2, −*z*+1; (iii) *x*−1, *y*, *z*; (iv) *x*, *y*−1, *z*; (v) −*x*+1, *y*−1/2, −*z*.
